# Impact of artificial urinary sphincter erosion in the reimplantation of the device

**DOI:** 10.1590/S1677-5538.IBJU.2022.0089.1

**Published:** 2022-07-19

**Authors:** André G. Cavalcanti

**Affiliations:** 1 Departamento de Cirurgia Geral e Especializada Universidade Federal do Estado do Rio de Janeiro Rio de Janeiro RJ Brasil Departamento de Cirurgia Geral e Especializada, Universidade Federal do Estado do Rio de Janeiro - UNIRIO, Rio de Janeiro, RJ, Brasil

## COMMENT

In this article, the authors assess the clinical presentation of patients with urethral cuff erosion after the implant of an Artificial Urinary Sphincter (AUS) and also attempt to establish risk factors in this patient population ( 1 ). As well pointed out by the authors, this complication has an extreme impact on the treatment of these patients, both in the acute phase, associated with the inflammatory and infectious condition, and late related to the reimplantation of the device. Replantation of AUS after erosion is certainly one of the most challenging conditions in the management of male urinary incontinence. Despite the literature describing statistical data and risk factors for erosion, these hardly describe details about the evolution characteristics of this process. As observed by the authors, most patients present with inflammatory signs in the scrotum, but a significant part of patients (about 1/3) do not present these symptoms, and will exclusively have urinary symptoms (obstruction or incontinence relapse) ( 2 ). This is an important finding because even in the absence of inflammatory signs, erosion should be suspected when there are urinary symptoms, whether obstructive or incontinence relapse. In the case of obstructive symptoms, it is important to analyze whether there is a reference to urethral stenosis or urethro-vesical anastomosis in the patient’s clinical history for the differential diagnosis. In the case of incontinence relapse, it is usually more acute when compared to other causes such as urethral atrophy. As well demonstrated in the study, these symptoms can present in combination. In the study, inflammatory symptoms are more associated with obstruction, perhaps due to greater urine leakage, a fact that also justifies lower rates of the combination of incontinence relapse and inflammation. The presence of radiotherapy was more common in patients with erosion when compared to those without erosion, as well as hypertension, coronary heart disease and smoking. The identification of risk factors is essential for patient consent, as well as for technical interventions to be taken to prevent the problem at the time of implantation of the prosthesis. The authors did not demonstrate previous procedures such as urethroplasty as risk factors in this study, but in our opinion, all patients who have an established impact on urethral vascularization a risk factor for cuff erosion. In the specific case of urethroplasty, we should try to preserve the urethra vascularization in all patients who are at risk of developing postoperative incontinence. Early recognition of cuff erosion is critical for an early approach that is likely to be associated with less urethral damage.
